# The Treatment of Fractures from a Common-Sense Point of View

**Published:** 1907-08-17

**Authors:** 


### Points in Surgery.

### THE TREATMENT OF FRACTURES FROM A COMMON-SENSE POINT OF VIEW.

### GENERAL CONSIDERATION.

The diagnosis of the presence of a fracture of a long bone is often a very easy matter. In fractures of the tibia, for instance, the nature of the lesion is obvious, on account of the unnatural mobility of the limb and the complete loss of its function, while the superficial position of the bone enables crepitus to be obtained easily and the ends of the fragments to be palpated. But even in the case of the tibia there are exceptions to this; and the writer remembers a case in which a fracture of the tibia was entirely unsuspected because the fibula, which was intact, supported the fragments and kept them in position. When the patient was walking out of the surgery the fibula gave way, and he fell to the ground; and the nature of his injury was then obvious.

On the other hand the diagnosis of fractures of long bones which are deep-seated and well covered with muscles may present some of the most difficult problems in surgery, unless a radiographic examination of the injured part is made. It has been said that the introduction of radiography has made the rising generation of medical practitioners careless in trying to diagnose this class of injury, and that the clinical instinct of the modern surgeon is less than that of his predecessors, who had to rely on their senses of sight and of touch alone. It need hardly be said that the knowledge that an absolute diagnosis can be made with the $x$-rays does not excuse carelessness. The surgeon must attempt to arrive at a clinical diagnosis; but there will always be cases that will defy the most careful examination, and wherever there is the least shadow of doubt as to the nature of the injury a radiogram should be taken.

### The Value of $x$-ray Examination.

This is, of course, an easy matter in London, especially in the great hospitals which are provided with a special department for this purpose alone. This is also the case in most provincial hospitals, whose authorities are willing to allow practitioners residing in the neighbourhood to bring up patients for radiographic examination. Medical men who have an extensive surgical practice at some distance from a provincial hospital would do well to provide themselves with a private radiographic apparatus. It is no exaggeration to say that they would find themselves well repaid for the initial outlay, which is not excessive. $X$-rays have taught us several lessons which should humble our pride, and were brought home by the evidence of several famous surgeons in a recent trial for surgical *mal praxis*. They stated that the diagnosis of fractures, made after the most careful clinical examination, was repeatedly found to be a wrong one when the case was submitted to radiography; and further, that in cases examined by the $x$-rays the ends of the bone were often found to be in a bad position, although they had fondly imagined the result to be an excellent one. The diagnosis of the site of a fracture by $x$-rays is scientifically accurate; and the writer lays so much stress on the importance of employing them, because it is practically impossible to obtain a satisfactory result in cases of fracture without an accurate diagnosis.

Another point which is often overlooked in dealing with fractures is the value of giving an anæsthetic, both in elucidating the diagnosis and in enabling the surgeon to get the fragments into position. It overcomes the muscular spasm which is caused by the pain of the injury, as anyone who has ever attempted to arrive at a diagnosis of the site of a fracture in the upper half of the femur is aware. To set a fracture without an anæsthetic not only causes the patient excruciating pain, but often allows no certainty of a good result. When, however, the muscles are quite relaxed, the problem is a simple one. Before giving the anæsthetic the various forms of apparatus which may be required should be in readiness. For instance, in dealing with a fracture of the femur, a sufficient number of pieces of strapping should be prepared for extension purposes, while a Hodgen's splint and a Liston's long splint should be in readiness, so that either may be immediately applied according as the exigencies of the case demand. The patient will be much comforted on coming round from the anæsthetic to find that the injured limb is comparatively speaking comfortable, and that his pain is relieved.

Thus the administration of an anæsthetic is of great value in assisting the first two manipulations in the treatment of simple fractures: they are (1) reduction, or the bringing of the ends of the bone level with each other; (2) coaptation, or the setting of the ends in accurate apposition. There are very few cases in which the overlapping ends of the bone cannot be reduced single handed; though occasionally it may be necessary to have an assistant to exert counter-extension by opposing the extending force applied by the surgeon. Extension should be made by taking a firm grasp of the limb well below the seat of fracture and pulling steadily in the long axis of the bone. It is especially important that extension should be applied gradually, if for any reason it has been decided not to give an anæsthetic. The effect of extension applied in this way is to tire out the muscles and overcome their resistance. Sharp or sudden movements tend to increase the muscular spasm, and the consequent contraction defeats the surgeon's object.

No time should be lost in reducing a fracture after the accident has occurred. Adequate coaptation of the fragments is more easily effected before the inevitable effusion which is caused by laceration of the tissues can become considerable, and, moreover, the sooner reduction is effected, the less effusion will there ultimately be.

